# 色谱分析中样品前处理技术的发展动态

**DOI:** 10.3724/SP.J.1123.2020.05011

**Published:** 2021-01-08

**Authors:** Weini HUANG, Zian LIN

**Affiliations:** 食品安全与生物分析教育部重点实验室, 福建省食品安全分析与检测重点实验室, 福州大学化学学院, 福建 福州 350116; 食品安全与生物分析教育部重点实验室, 福建省食品安全分析与检测重点实验室, 福州大学化学学院, 福建 福州 350116

作为现代分析领域的重要手段之一,色谱可用于食品、环境、生物等复杂样品的定性与定量分析。而样品前处理是获得理想色谱分析结果的必要前提,它可有效消除基质干扰、浓缩目标分析物、改善分离效能以及延长色谱仪器使用寿命。本文主要依据2020年4-5月Anal Chem新发表和在线录用的有关论文,讨论色谱分析中涉及样品前处理技术方面的最新发展动态。

## 1 相分离萃取技术

相分离萃取技术是基于目标分析物在萃取相和样品之间的动态分配过程,达到样品富集、净化的前处理效果,主要包含由固相萃取(SPE)和液相萃取发展来的多种分离萃取形式。

SPE作为经典的样品前处理技术,正在逐步向微型化、集成一体化发展。近期,吉林大学顾景凯课题组^[1]^采用Oasis HLB μElution微量萃取96孔盘对大鼠血浆、尿液和粪便中的超高相对分子质量聚环氧乙烷(1700 kDa)进行固相萃取,通过反相液相色谱-三重四极杆/飞行时间质谱进行准确定量。微固相萃取技术使用高容量吸附剂且将淋洗体积降至25 μL,浓缩程度高,为后续分析实现高回收率和低基质效应奠定了基础。该研究填补了生物样品中聚环氧乙烷的定量研究空白,进一步完善了口服聚环氧乙烷的临床代谢和排泄信息,也为分析生物样品中具有超高相对分子质量和宽分散性的聚合物提供了新方向。

韩国科学技术研究院Cheolju Lee课题组^[2]^设计了一种封装固相萃取装置,开发了集成富集N端肽的方法(iNrich)。首先,将稀释后的蛋白质样品装载在聚丙烯核磁共振管盖内侧,对氨基标记时同步预处理SPE柱。将管盖组装到SPE柱上端,同时塞住底部,形成密封固相萃取柱环境。胰蛋白酶等反应物可通过开盖添加,混匀。富集完成后取下两端盖塞,洗脱液经LC-MS/MS分析。该分析方法构建了N端肽蛋白组学平台,从100 μg人类细胞蛋白质中鉴定出约5000种N端肽。SPE其他应用进展还包括发展新型吸附剂,所发展的新型吸附剂可成功提取分析尿样中非甾体抗炎药和高效拆分色氨酸对映异构体^[3,4]^。

非平衡固相微萃取技术集采样、萃取、浓缩、进样于一体,分析检测速度得到极大提升。西班牙萨拉戈萨大学Cristina Nerín课题组^[5]^利用食品模拟物进行食品接触材料(FCM)迁移测试实验,发展了浸入式固相微萃取(DI-SPME)方法富集再生聚烯烃中的非故意添加物质(NIAS),选择FCM中常见的35种参考标准品作为检测对象,结合GC-MS评估该法作为FCM中非目标筛选NIAS的潜力。该研究优化选择DVB/CAR/PDMS萃取涂层,确定DI-SPME重要因子并利用响应面法优化实验参数。结果表明经DI-SPME-GC-MS分析,除了胺和二醇的LOD较高外,其余物质的LOD均在0.1~14.1 μg/kg。此外,国内学者郭佃顺等^[6]^将微波辅助合成类二氧芑连接结构的共价有机骨架材料(TH-COF)作为SPME纤维涂层,应用于分析水样中的全氟烷基化合物,效果良好。

无独有偶,在液相萃取方面,新加坡国立大学Hian Kee Lee课题组^[7]^采用聚丙烯膜袋液相微萃取(MBA-LPME)富集水样中的糖皮质激素,选择正辛醇和表面活性剂十二烷基硫酸钠混合溶液作萃取剂,富集系数为32~189。用UHPLC-MS分析,检出限为0.0331~0.166 ng/mL。该微萃取装置可作为蠕动泵和自动进样器的中间连接部件,搭建全自动集成分析平台,对日常检测水质污染物具有重要意义。捷克科学院Pavel Kubáň课题组^[8]^则是发展了一种新型一次性中空纤维液相微萃取装置(HF-LPME),可在线耦合毛细管电泳(CE)分析生物样品。多孔HF首先浸渍在有机溶液中形成支撑液膜,注入5 μL受体溶液,CE小瓶注入供体溶液。引入锥形聚丙烯微量注射器吸头固定HF于CE小瓶中的位置,同时充当管状电极和毛细管的导向器。搅拌萃取完毕后,直接从HF腔内进样分析得到的受体溶液。可采用多孔板形式实现快速、简单、自动化分析样品。该方法成功应用于人类尿液和干血斑(DBS)样品中基础药物的检测,富集因子为37~84,检出限为0.7~1.55 μg/L。值得注意的是,DBS洗脱可与HF-LPME富集同时进行,具有直接分析DBS样品的巨大潜力。此外,西班牙学者^[9]^利用3D打印技术自制一种螺旋状HF-LPME装置,结合CO_2_泡腾可实现饮用水中消毒副产物的高倍富集。

浊点萃取法是以表面活性剂胶束水溶液的溶解性和浊点现象为基础,通过改变温度、pH等实验参数引发相分离。广州大学闫兵课题组^[10]^发展了浊点萃取法(CPE)富集环境水样中金属基纳米颗粒(M-NPs),结合LC-ICP MS检测技术,创新性实现M-NPs中金属硫化物纳米颗粒(MS-NPs)的定量分析。水样中M-NPs首先富集浓缩于富含表面活性剂0.15%(m/v)Triton X-114的有机相中,期间纳米颗粒的形态不变。随后,添加去水双(对-磺酰苯基)苯基膦化二钾(BSPP)溶液可使胶束消失,解决分析样品的高黏问题,同时作非MS-NPs的解离剂。通过选择LC-ICP MS系统的不同分离模式,测得M-NPs总量(未经色谱柱分离)和非MS-NPs总量(经色谱柱分离),从而计算得MS-NPs总量。结果发现Ag_2_S-NP和ZnS-NP检出限分别为8和15 ng/L,此技术在环境中监测痕量M-NPs有广泛的应用前景。

## 2 衍生化

衍生化可显著提高检测对象的可分析性,有助于应对分离分析同分异构体的挑战。奥地利学者Christina Troyer课题组^[11]^利用Gerstel MPS2双轨样品制备系统对3种*N*-乙酰己糖胺异构体进行在线自动衍生化,通过软件控制,样品经前处理制备完成后可自动进样至气相色谱分离。经烷氧基化和三甲基硅烷化两步在线衍生,开发GC-MS/MS、GC-TOFMS两种检测方式均可分离并定量测定3种*N*-乙酰基糖胺立体异构体,并成功应用于青霉菌实际样中*N*-乙酰葡糖胺的定量分析。

建立高通量分析平台,丰富化合物数据库是分析人员的追求。近日,加州大学戴维斯分校Oliver Fiehn课题组与武汉大学冯钰锜课题组^[12]^合作发展了一种基于2-二甲氨基乙胺(DMED)的化学衍生策略,解决羟基脂肪酸酯(FAHFA)在负电离模式质谱法中灵敏度低的问题。该法通过用正离子化的叔胺基团标记FAHFA,极大地提高了正离子模式下电喷雾质谱的电离效率,经UPLC-MS/MS分析,开发了涵盖264种FAHFA的质谱数据库。此外,清华大学瑕瑜课题组^[13]^通过在LC与ESI间设置透明的流动微反应器,基于Paternò-Büchi (PB)反应进行脂质的在线光化学衍生。同时,改进流动相组成,建立了RPLC-PB-MS/MS鉴定不同类别脂质的工作流程。该法可识别磷脂异构体中的碳碳双键位置,具有大规模进行脂质定性定量分析的潜力。

## 3 其他

干血斑分析是将血液点在滤纸上并使之干燥,再运到实验室分析的技术,利用远程采集样品解决偏远地区分析检测能力弱的问题。基于此技术,德国Paul W. Elsinghorst课题组^[14]^开发了干萃取点(DES)实现霉菌毒素远程送检的方法(myco DES)。首先,采用QuEChERS方法快速萃取谷物类食品中霉菌毒素,在乙腈溶液中添加柠檬酸盐盐析萃取分离相,添加同位素内标,通过毛细管在DES纸卡上点样,干燥封装。随后模拟欠发达地区气候运输,最后在中心实验室重新提取DES,可在LC-MS/MS实现定量分析。结果表明在热带气候条件下储存4周后仍可定量检测霉菌毒素安全水平。

加拿大麦克马斯特大学的Yu Lu课题组^[15]^设计了一锅式单管处理纳米蛋白组学样品(NanoTPOT),在单个Eppendorf Lobind低蛋白吸附管中进行细胞裂解、蛋白质酶解和标记串联质谱标签TMT。优化NanoTOPT工作流程,消除了样品脱盐需要,后由LC-MS/MS进行定量分析。该法应用于经二硫苏糖醇处理后的Hela细胞变化,结果发现1 μg裂解物可在TMT 10-plex实验中鉴定并定量6935种蛋白质,充分体现了NanoTOPT在纳米蛋白组学分析中的发展前景。

## 4 结语

以上是色谱研究中样品前处理技术的最新成果列举,其分析对象与检测技术丰富多样,但总体表现出了样品前处理装置更加微型化、智能化的发展趋势。联用色谱-质谱分析方法,样品处理技术将会在进行高通量快速筛查化合物中扮演越来越重要的角色。
